# Laser Patterning of Aligned Carbon Nanotubes Arrays: Morphology, Surface Structure, and Interaction with Terahertz Radiation

**DOI:** 10.3390/ma14123275

**Published:** 2021-06-14

**Authors:** Olga V. Sedelnikova, Dmitriy V. Gorodetskiy, Alexander G. Kurenya, Kseniya I. Baskakova, Elena V. Shlyakhova, Anna A. Makarova, Gleb V. Gorokhov, Dzmitry S. Bychanok, Polina P. Kuzhir, Sergey A. Maksimenko, Lyubov G. Bulusheva, Alexander V. Okotrub

**Affiliations:** 1 Nikolaev Institute of Inorganic Chemistry SB RAS, 630090 Novosibirsk, Russia; gordim2005@yandex.ru (D.V.G.); kurenyaag@niic.sbras.ru (A.G.K.); baskakova@niic.nsc.ru (K.I.B.); ShlyakhovaEV@niic.sbras.ru (E.V.S.); bul@niic.nsc.ru (L.G.B.); 2 Physical Chemistry, Institute of Chemistry and Biochemistry, Free University of Berlin, 14195 Berlin, Germany; anna.makarova@fu-berlin.de; 3 Institute for Nuclear Problems, Belarusian State University, 11 Bobruiskaya str., 220006 Minsk, Belarus; glebgorokhov@yandex.ru (G.V.G.); dzmitrybychanok@yandex.by (D.S.B.); polina.kuzhir@uef.fi (P.P.K.); sergey.maksimenko@gmail.com (S.A.M.); 4 Physics Faculty, Vilnius University, Sauletekio 9, LT-10222 Vilnius, Lithuania; 5 Institute of Photonics, University of Eastern Finland, Yliopistokatu 7, FI-80101 Joensuu, Finland

**Keywords:** CNT arrays, laser patterning, SEM, XPS, NEXAFS, Raman, terahertz elements

## Abstract

The patterning of arrays of aligned multi-walled carbon nanotubes (MWCNTs) allows creating metastructures for terahertz (THz) applications. Here, the strips and columns from MWCNTs vertically grown on silicon substrates are prepared using CO_2_ laser treatment. The tops of the patterned arrays are flat when the laser power is between 15 and 22 W, and craters appear there with increasing power. Laser treatment does not destroy the alignment of MWCNTs while removing their poorly ordered external layers. The products of oxidative destruction of these layers deposit on the surfaces of newly produced arrays. The oxygen groups resulting from the CO_2_ laser treatment improve the wettability of nanotube arrays with an epoxy resin. We show that the patterned MWCNT arrays absorb the THz radiation more strongly than the as-synthesized arrays. Moreover, the pattern influences the frequency behavior of the absorbance.

## 1. Introduction

Carbon nanotubes (CNTs) possess exceptionally high terahertz (THz) conductivity and, therefore, can replace the bulk metallic components and nanowires in nanoelectronics [1], communications [2,3], medicine [4], and many others [5,6]. However, the use of individual CNTs is limited in practice because of the sensitive dependence of nanotube properties on the structural configuration, which can be random, despite certain progress in the synthesis of CNTs with specific geometry [7,8]. Today, bulk materials based on CNTs are drawing the attention of researchers. Arrays of CNTs stand out among such materials for several reasons, the main one being their morphology. Arrays, composed of packed side-by-side parallel CNTs of equal length and close diameter, have electrical conductivity closest to individual CNTs due to the minor contribution of the CNT-CNT tunneling effect in charge transport phenomena [9].

The ordering of conducting microelements allows controlling and manipulating the incident THz radiation [10]. Transfer of this ideology to CNTs requires the development of a method for their precise positioning [11]. Only a few techniques allow obtaining patterned growth of aligned CNT areas, such as templating of a substrate to define the patterns of catalytic layers [12,13] or using lithography [14,15,16] followed by the conventional chemical vapor deposition (CVD) synthesis of nanotubes. Another technique is the post-growth treatment based on the ablation of an array by a focused laser beam. Applications of femtosecond [17,18,19,20,21] and nanosecond [17,22,23,24,25,26,27] pulsed and continuous [17,28,29,30,31] lasers were considered. Due to the high longitudinal thermal conductivity of nanotubes and significantly suppressed radial heat transfer [32,33], the patterning of arrays with short-pulse lasers does not provide a decisive benefit in the quality of cut edges [17] as it is used for the ablation of dense targets [34]. With the need to scan a large area and reduce the overall cost of material, a high-power continuous laser seems to be the best choice for the patterning technology of CNT arrays.

Although the idea to use focused laser irradiation for the microstructuring of CNT arrays is not new, the quality of engraved elements surfaces and the possible transformation of preserved nanotubes after treatment are understudied. Commonly used characterization methods are scanning electron microscopy (SEM) and Raman spectroscopy. The former method does not reflect changes in the CNT walls, and the latter summarizes the spectra of the nanotubes located within a thickness of 500 nm below the array surface. As a result, the surface state of engraved array areas remains to be hidden.

In this study, we used continuous CO_2_ lasers in the 15–38 W range to pattern arrays of aligned CNTs grown using an aerosol-assistant catalytic CVD technique. The methods of SEM, surface-probing X-ray photoelectron spectroscopy (XPS), bulk-probing near-edge X-ray absorption fine structure (NEXAFS) spectroscopy, and micro-Raman spectroscopy were used to study the effect of laser power on the structure and composition of CNTs forming arrays. We determined the laser power that does not lead to significant modifications of the CNTs and prepared arrays of ordered strips and columns of aligned CNTs. The effect of the pattern on the THz response of aligned CNT array was revealed.

## 2. Materials and Methods

Vertically aligned multi-walled CNTs (MWCNTs) arrays of uniform height ca. 300 μm were grown using a CVD method on silicon wafers with (100) orientation [35]. A reaction mixture composed of 4 wt % of ferrocene and 96 wt % of toluene was injected into a tubular reactor heated at 800 °C. An analysis of the TEM images (a JEOL-2010 microscope, JEOL Ltd., Tokyo, Japan; Figure S1a in Supplementary Materials) of the nanotubes determined the average values of ca. 20 nm for the outer diameter (Figure S1b in Supplementary Materials), ca. 8 nm for the inner diameter (Figure S1c in Supplementary Materials), and about 6 nm for wall thickness. The estimated number of layers in the MWCNTs was 17. High-resolution TEM images showed good-quality internal layers aligned along the nanotube cavity and poorer ordering of the outer layers (Figure S1 d in Supplementary Materials).

The arrays were patterned by a laser engraver (Winseal, Guangzhou, China) equipped with a continuous CO_2_ laser with a wavelength of 10.4 μm. The method, described in detail elsewhere [36,37], is based on the oxidation of nanotubes under infrared radiation. Briefly, the laser beam with a diameter of ca. 50 μm was focused on the top surface of an array. Strips of MWCNT arrays were obtained through line-by-line scanning of the MWCNT samples. To create the columns, the samples were rescanned in a cross-direction. The scanning speed was kept at 17 mm/s, and the grating period was about 300 μm. The laser output power was ca. 15, 22, 30, and 38 W. As a result, four patterned samples composed of spaced columns of MWCNTs were obtained, denoted as Sample A, B, C, and D, respectively. During laser treatment, white-light emission from irradiated areas was observed. This effect can be attributed to laser-induced incandescence associated with invasive burnout of individual CNTs [30].

The morphology of the engraved arrays was studied by SEM on a Hitachi S-3400N microscope (Hitachi Ltd., Tokyo, Japan).

Room-temperature Raman scattering spectra were acquired using a LabRAM HR Evolution spectrometer (Horiba, Kyoto, Japan) using a 514 nm excitation of Ar^+^-laser, normalized to an intensity of 1572 cm^−1^ (G peak).

The elemental compositions of the initial engraved arrays were determined by energy-dispersive X-ray (EDX) spectroscopy on a Hitachi S-3400N scanning microscope (Hitachi Ltd., Tokyo, Japan) and XPS. The chemical state of the carbon was studied by NEXAFS. XPS and NEXAFS spectra were recorded at the synchrotron radiation facility BESSY II, Helmholtz-Zentrum Berlin, using radiation from the Russian-German dipole (RGBL dipole) beamline. Arrays were placed into a chamber maintained at a pressure of 10^−9^ mbar. The XPS spectra were measured at a monochromatic radiation of 830 eV. The electrons emitted normally to the sample surface were collected, and the angle between the incident radiation and analyzer was 55°. After subtracting a Shirley background, the C 1s and O 1s XPS spectra were fitted using Gaussian/Lorentzian functions within Casa XPS software (version 2.3.15, Casa Software Ltd, Teignmouth, UK). The NEXAFS spectra near the C K-edge were recorded in the total-electron yield.

To demonstrate the prospects of microstructured MWCNT arrays for THz technologies, we investigated two samples composed of spaced strips and columns by scanning with aligned MWCNTs arrays (denoted as Samples 1 and 2). The engraving conditions included: scanning speed of 17 mm/s, laser power of 15 W, grating period of ca. 250 μm, and engraved element height of ca. 170 μm. The engraved strips were covered with epoxy resin (Crystal 76) under vacuum conditions. The resin mixed with hardener was applied to the MWCNT array with a pipette until the sample was completely soaked with resin. The excess resin was allowed to drip down from the inclined samples under evacuation. The THz mirror reflection coefficient was measured using a TSPEC time-domain spectrometer (EKSPLA, Vilnius, Lithuania) in a 0.1–1.4 THz frequency range.

## 3. Results and Discussion

### 3.1. Effect of the Laser Power

**Figure 1:**
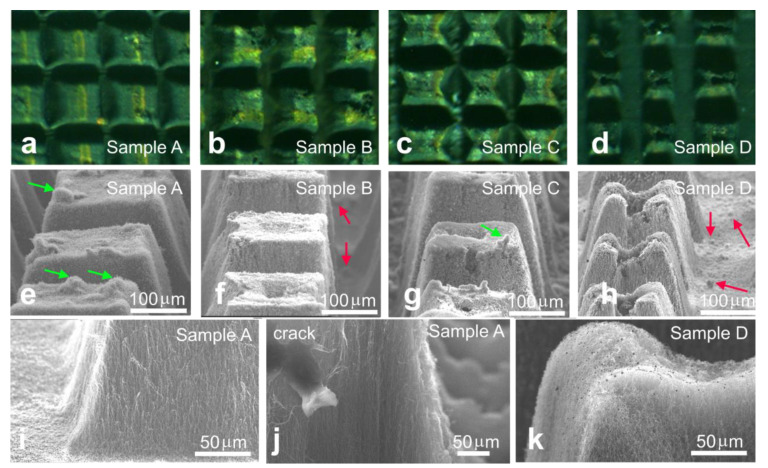
Optical and SEM images of Sample A (**a** and **e**,**i**,**j**), Sample B (**b** and **f**), Sample C (**c** and **g**), and Sample D (**d** and **h**,**k**).

We first analyzed the effect of laser power on the array structure. Four samples with similar columnar patterns were engraved with the 15, 22, 30, and 38 W laser beams (denoted as Samples A, B, C, and D, respectively). The optical images showed that all arrays were burned down to the substrates (Figure 1a–d). The increase in laser power enhanced the deviation in the columns from parallelepipeds and changed their top surface. In particular, the tops of the columns of Sample A were flat (Figure 1e). Samples B and C had small holes in the column centers (Figure 1f,g). The columns in Sample D had deep craters with ragged edges (Figure 1h). There were round-shaped carbonaceous material contaminations (shown by green and red arrows in Figure 1e,f,g,h) presented on vertexes of Sample A columns, whereas for Samples B, C, and D, they were found mainly on the silicon substrates. The side surface of columns in Sample A contained shortened nanotubes, whose initial length before engraving was an array height of ~300 μm (Figure 1i). The nanotubes were generally aligned perpendicular to the substrate. The image taken from a crack of one of the columns showed a good ordering of the interior MWCNTs (Figure 1j), the same as the initial array (see Ref. [4]). Moreover, there was a poorly structured surface layer of ~5–10 μm in width. This meant that the interior regions of arrays were unaffected by the 15 W beam. Burning holes in the column centers under processing with higher laser power suggested that even the internal MWCNTs, which were not directly exposed to the beam, were modified during the engraving. Moreover, the morphology of Sample D surface layer changed. Nanotubes were entangled with each other, forming a cotton layer instead of an aligned structure (Figure 1k). The dark nanoparticles distributed on the surface were probably de-encapsulated catalyst nanoparticles.

**Figure 2:**
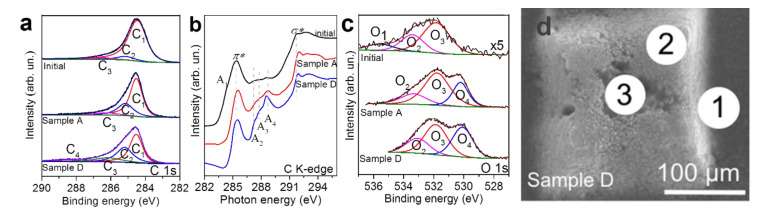
XPS C 1s (**a**), NEXAFS C K-edge (**b**), and XPS O 1s (**c**) spectra of initial and engraved MWCNT arrays. SEM image of a column of Sample D (**d**). Circles indicate regions used for EDX analysis.

The XPS method was used to reveal differences in the chemical states of atoms located on the surface of columns treated by the 15 and 38 W laser beams (Figure 2a). The survey XPS spectra in the range of 0–830 eV (not shown) were recorded to identify the surface elements present in the samples. For the initial array, the major constituent was carbon (~98 at %), with a minor contribution from oxygen (~2 at %). Few peaks fit the XPS C 1s spectra. The dominant component located at 284.5 eV (peak C1) corresponds to the *sp*^2^-hybridized carbon. For this feature, the full width at half maximum (FWHM) was ca. 1.4 eV (Figure 2a), which significantly exceeds the value for highly oriented pyrolytic graphite (ca. 0.9 eV). During the BESSY II experiments, the samples were attached to the spectrometer holder so that the spectrum of the initial array was collected from its top surface. The probing depth of XPS acquired using radiation of 830 eV was ca. 3 nm [38], indicating rather high disorientation of MWCNTs in the vertex of the untreated array. The weak high-energy shoulder consisted of peaks located at 285.2 eV (peak C2) and 286.4 eV (peak C3). The former component is due to contributions from *sp*^3^-hybridized carbons [39] or carbon atoms linked with –CO species [40], and the latter is due to different oxidized forms of carbon (C–O–C or C–OH) [40,41,42].

The NEXAFS C K-edge spectrum of the initial array was dominated by two resonances (Figure 2b) assigned to the 1s electron transition to unoccupied π* and σ* orbitals of the *sp*^2^-hybridized carbon structure (positioned at 285.4 and 291.8 eV, respectively). Shoulder A1 on the low-energy side of π* resonance can be associated with the states of disordered carbon [43]. Weak peaks at ~287.1 eV (A2), 287.9 eV (A3), and 288.6 eV (A4) indicated that few covalent bonds of carbon with heteroatoms were in agreement with the XPS data. According to previous works [40,43,44], peaks A2 and A3 correspond to carbon bonded with hydroxyl and ethers and/or aliphatic groups, peak A4 can be assigned either to the transitions to π* states of a carboxyl group or σ* states of C–H bonds from amorphous carbon species.

According to XPS data, the engraved samples obtained at the lowest and highest laser power (Samples A and D, respectively) contained about 14 at % of oxygen and about 4 at % of iron. The emergence of iron occurs due to the transport of encapsulated catalyst nanoparticles from the cavities of nanotubes under high-energy exposure. A similar effect was previously obtained for plasma-treated MWCNTs [45], single-walled CNTs filled with sulfur [46], and MWCNTs under femtosecond laser radiation [18]. The XPS C1s spectra are also governed by *sp*^2^-hybridized carbons (peak C1) with the FWHM of ca. 0.9 eV (Figure 2a), indicating a rather high quality of MWCNTs. The NEXAFS data confirm this. The π* resonance at 285.4 eV of Samples A and D was quite narrow without the shoulder, and the σ* resonance at 291.8 eV was sharpening. (Figure 2b). However, a significant contribution from *sp*^3^ carbon was observed. Firstly, the C1s spectra contained notable peaks at 285.5 eV (peak C2). The ratio of sp^3^/sp^2^ carbon deduced from the areas of the components C2 and C1 increased from a value of 1/12 for the initial array to two-fifths for Sample A and three-fifths for Sample D. Secondly, the C K-edge spectra of the engraved samples showed a decrease in the π*/σ* ratio compared with that of the initial array and prominent resonance at 288.6 eV. The latter should be mainly related to C–H bonds due to a rather low intensity of carboxyl-related peak (peak C4) in the XPS C 1s spectra of the engraved samples. These findings suggest that in addition to well-ordered MWCNTs, Samples A and D consisted of amorphous-like carbonaceous components. The latter could be assigned to round-shaped particles observed in SEM images (Figure 1). In contrast, the initial array contained weakly functionalized nanotubes with disordered external layers in agreement with a recent study [47] and our TEM results (Supplementary Materials). Moreover, both the XPS and NEXAFS spectra indicated the presence of oxygen-containing moieties on the sample surface, namely, peaks C3 at 286.4 eV and C4 at 288.6 eV in the C 1s spectra and peaks A2 at 287.1 eV and A3 at 287.9 eV in the C K-edge spectra.

The consequent increase in the intensities of oxygen-related peaks in the carbon spectra with laser power was a good indicator of surface functionalization of Samples A and D. The XPS O 1s spectra helped determine the location of the moieties (Figure 2c). The spectrum of the initial array was fitted by three peaks positioned at 535.5 eV (peak O1), 533.5 eV (peak O2), and 531.8 eV (peak O3). These bands can be related to the adsorbed water (peak O1), ether (C–O–C), and hydroxyl (–OH) groups (peak O2), or the C=O bonds in the carboxyl group (peak O3). For the engraved samples, the contribution from adsorbed water was absent, during which the iron oxide peak O4 located at 530.2 eV appeared. The O3/O2 ratio decreased under engraving, indicating the different compositions of the surface groups in the initial array and the engraved samples. Considering the high thermal stability of MWCNTs, the prior oxidation of amorphous particles indicates that the oxygen-containing groups are located mainly on these contaminants.

Figure 2d shows the SEM image of a column from Sample D with marked regions (denoted as 1, 2, and 3) that were used to measure the local elemental composition with EDX spectroscopy. There are signals from carbon, silicon, oxygen, and iron, in agreement with the XPS data. The data collected from a side (point 1) show 86.6 at % of C, 10.9 at % of Si, 0.9 at % of Fe, and 1.4 at % of O. A flat region of vertex contains ca. 92.2 at % of C, 0.1 at % of Si, 2.2 at % of Fe, and 5.4 at % of O (point 2). The values for Fe and O are close to those (5.3 and 2.1 at %, respectively) found inside a crater (point 3). Based on these data, we propose that, air oxygen quickly leaves the reaction zone and interacts with MWCNT walls at elevated temperatures. This process is followed by the emission of CO_2_ and equalization of oxygen content through the whole sample. The signal from silicon was absent at point 3. Since the electrons escaped from a depth of about 5 μm, the EDX spectra probed the composition of the entire laser-affected zone of the nanotube array, while the previously discussed XPS method studied the surface layer of about 3 nm. The lower EDX oxygen concentration indicates stronger oxidation of the outermost nanotubes.

**Figure 3:**
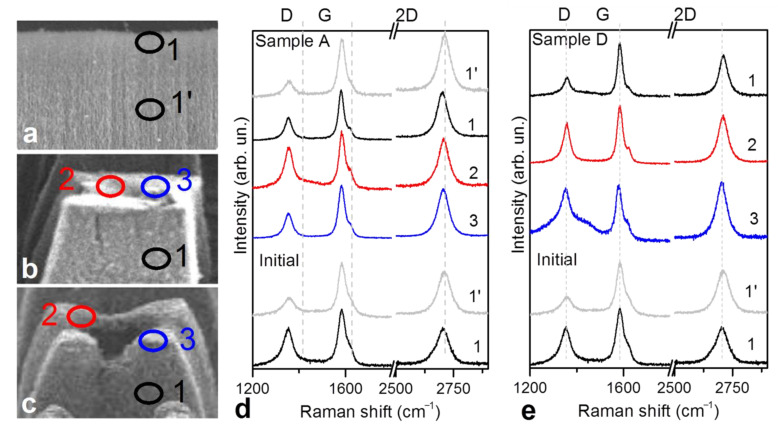
Points used to collect Raman spectra for initial MWCNT array (**a**), Sample A (**b**), and Sample D (**c**). Local Raman spectra of Sample A (**d**) and Sample D (**e**) compared with those of the initial array. Spectra are labeled by collection point explained in (**a**,**b**,**c**).

To further analyze the surface composition of the engraved samples, the local Raman spectra were collected from the points indicated in Figure 3a–c. For the initial sample, we compared spectra taken from the top surface (Figure 3, point 1) and a side crack of the array (Figure 3, point 1’). Both spectra have three prominent peaks located at 1351, 1572, and 2710 cm^−1^, which are related to the D, G, and 2D bands, respectively. The G band is a graphite E_2g_ mode, where neighboring atoms vibrate along to the graphitic sheet. The D band is due to a breathing mode of *sp*^2^ carbon rings. The 2D band, arising from the two-phonon process, is associated with the crystallinity of MWCNT walls. The spectra of the initial array acquired from points 1 and 1’ show different intensities of the D band (the *I*_D_/*I*_G_ value was ca. 0.7 and 0.4, Table 1) and 2D band (the *I*_2D_/*I*_G_ value was ca. 0.7 and 0.8, Table 1). Hence, the *sp*^2^ networks of MWCNTs located close to the array top were more disordered than in the internal nanotubes. This result agrees with the XPS and NEXAFS data.

The Raman data for the engraved samples also depend on the collection point (Figure 3d,e). The effect of laser treatment on the defectiveness of the *sp*^2^ carbon network can be estimated from the change of intensities of the D and 2D bands (Table 1). MWCNTs located on the side surface of columns exhibit the comparable (Sample D) or even better (Sample A) crystallinity of the walls than the nanotubes within the initial array. Therefore, when external nanotubes are not directly exposed to the laser beam, they remain unchanged. The exception is craters formed in the centers of columns of Sample D. The spectrum acquired from this position had an intense and broad D band and enhanced 2D band, contributed by the amorphous constituent and highly crystalline short MWCNTs, respectively [48]. The contribution from silicon substrate in the low-wave-number region (not shown) indicated that the crater was rather deep. The spectrum of Sample A collected at a column side crack had the lowest-intensity D band and the highest-intensity 2D band. This observation may indicate improvement in the internal nanotubes’ walls due to the engraving, in agreement with the recent results [20,21,24,25].

The results described above indicate that the CO_2_ laser treatment affects the MWCNTs located both on the surface of engraved elements and inside them. This can be explained as follows: absorption of the laser beam results in the oxidation of nanotubes and ablation of carbon clusters [22,34]. White-light emission of heating MWCNTs is observed, followed by the burnout of nanotubes directly exposed to a laser. The nanotubes located at the surface layer of the engraved elements are oxidized at defect sites. This process likely causes the partial burning of the disordered external layers of MWCNTs and amorphous carbon produced during sample synthesis. Moreover, the flow of heat air can cause the straightening of MWCNTs, decreasing their bending [24]. As a result, the sample purity and the crystallinity of nanotubes walls are improved compared with the MWCNTs from the initial array. After irradiating the surface, the ejected material is re-deposited as amorphous nanoparticles on columns and silicone substrate. When the laser moves away, the surface layer cools quickly due to convection by the surrounding gas molecules [31], broadband light emissions, and energy transfer inside the array. Although the CNT–CNT coupling decreases the thermal conductivity of the array [49], when laser power is high enough, inside engraved elements at some distance from the laser pass, the hot zone is formed [27]. Unlike the surface layer, the cooling of this zone is hampered due to its limiting interaction with air molecules and self-absorption of emitted light through heating the MWCNTs. As a result, the nanotubes located there react with oxygen trapped inside the array, further increasing local temperature. This process is supposed to be self-sustained until all oxygen moieties react with carbon. In particular, that could explain the formation of craters in the center of columns obtained using a 38 W laser.

### 3.2. THz Response of the Arrays

**Figure 4:**
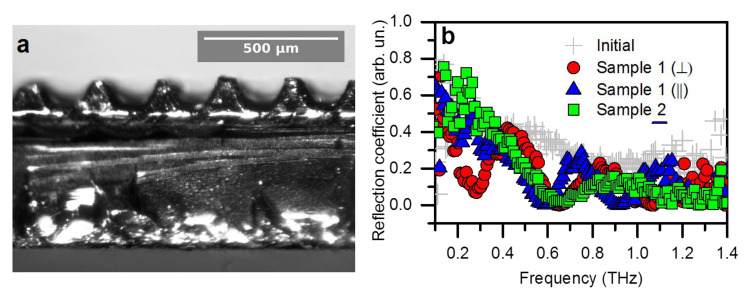
Optical image of Sample 1 (**a**). Reflection coefficients of Sample 1 and Sample 2 compared with epoxy-coated initial array of CNTs (**b**). Symbols ‖ and ⊥ correspond to the parallel and perpendicular orientation of engraved strips of Sample 1 relative to the polarization of the incident wave.

Previous investigations showed that CNTs have attractive intrinsic electromagnetic properties in the THz frequency range [50,51] and as a filler in dielectric matrices [52,53,54]. In particular, the properties of composites are strongly affected by the ordering of nanotubes [52,53] and surface functionalization [54]. The nano-structured arrays show a morphology-governed response in the optical region [15,16,21]. To test the suitability of the microstructured arrays of CNTs for THz applications, we fabricated two samples composed of engraved strips and columns (Samples 1 and 2) and measured their reflection coefficient in the frequency range of 0.1–1.4 THz. The patterning was performed at a laser power of 15 W to keep the engraved elements’ interior intact. Because engraved arrays of MWCNTs are fragile, the samples were uniformly covered by a thin epoxy layer (ca. 70–90 μm), as can be seen from the optical image in Figure 4a. The structural characterization results discussed above reveal strong oxidation of the MWCNTs’ surface after the treatment. Despite some deterioration in THz conductivity [54], the oxidized MWCNTs had an advantage over the initial ones due to improved wettability of the nanotube arrays with an epoxy resin, caused by the direct chemical interaction of MWCNTs with the matrix molecule through different oxygen-containing functional groups [55,56].

As shown in Figure 4b, the initial epoxy-covered array reflected from 30% to 80% of the incident light power in the frequency range of 0.3–1.4 THz, indicating that the covered array was no longer a perfect absorber. This effect was caused by an interference of waves reflected from the front and back sides of the epoxy layer. For the patterned samples, the portion of reflected radiation decreased to about 1–5% above 1 THz. This indicates an increase in the contribution of absorption in our samples compared with the non-structured epoxy-coated array. The spectra of the engraved samples showed interference oscillations. Because they were absent in the coated initial array, the oscillations arose due to strips and columns of MWCNTs arrays in Samples 1 and 2. Moreover, the response of Sample 1 depended on the strips’ orientation relative to the polarization direction of the incident wave. The spectra of the perpendicularly oriented pattern and the columns are alike. This suggests that the polarization-dependent properties can be assigned to the presence of a conduction path along the strips, while the conductivity between neighboring strips or columns behaves in a tunneling manner. A polarization-dependent THz response is essential for bio detectors [57]. Therefore, patterned arrays of CNTs can promote the design of advanced THz devices, which can distinguish polar biomolecules more accurately.

## 4. Conclusions

In conclusion, we synthesized aligned arrays of MWCNTs and patterned them with strips and columns through CO_2_ laser treatment. A comprehensive investigation of morphology and surface state revealed that the engraved elements consisted of aligned MWCNTs with improved crystallinity of their walls due to the oxidation of the outermost disordered layers. Changing the laser power controlled the microstructure of the engraved elements. In particular, the increase in the laser power from 15 to 38 W caused the formation of deep craters on the flat columns’ vertexes. The insufficient convection by surrounding gas molecules and self-absorption of incandescent light of heated MWCNTs was the origin of oxidation of the MWCNT array areas, which were not directly exposed by the laser beam. Owing to the improved wettability, the treated surface of MWCNTs robustly bound to epoxy molecules. This allowed the fabrication of lasting materials composed of epoxy-coated patterned MWCNTs arrays, which act as frequency-selective absorption elements in the THz frequency range. The results presented in this work develop a technological basis for THz metasurface production.

## Figures and Tables

**Table 1:** Ratios of Raman band intensities for the initial array, Sample A, and Sample D determined from the spectra collected at different array points as indicated in Figure 3.

Sample	Initial Array		Sample A				Sample D		
point	1	1’	1	2	3	1’	1	2	3
*I*_D_/*I*_G_	0.66	0.37	0.47	0.66	0.48	0.26	0.36	0.71	0.92
*I*_2D_/*I*_G_	0.69	0.81	0.95	0.72	0.95	1.14	0.78	0.82	1.07

## Data Availability

The data presented in this study are available on request from the corresponding authors.

## Supplementary Materials

The following are available online at https://www.mdpi.com/article/10.3390/ma14123275/s1, Figure S1: TEM image of MWCNTs from an array (**a**). Outer (**b**) and inner (**c**) diameter distribution of MWCNTs calculated from (**a**). HR TEM image of MWCNTs (**d**).

## References

[1] Lawler N.B., Ho D., Evans C.W., Wallaceb V.P., Iyer K.S. (2020). Convergence of terahertz radiation and nanotechnology. J. Mater. Chem. C.

[2] Rosenau da Costa M., Kibis O.V., Portnoi M.E. (2009). Carbon nanotubes as a basis for terahertz emitters and detectors. Microelectronics J..

[3] Akyildiz I.F., Jornet J.M. (2010). Electromagnetic wireless nanosensor networks. Nano Commun. Netw..

[4] Wang R., Xie L., Hameed S., Wang C., Ying Y. (2018). Mechanisms and applications of carbon nanotubes in terahertz devices: A review. Carbon.

[5] Batrakov K.G., Kibis O.V., Kuzhir P.P., Rosenau da Costa M., Portnoid M.E. (2010). Terahertz processes in carbon nanotubes. J. Nanophotonics.

[6] Portnoi M.E., Kibisb O.V., Rosenau da Costa M. (2008). Terahertz applications of carbon nanotubes. Superlattices Microstruct..

[7] Chen Y., Zhang Y., Hu Y., Kang L., Zhang S., Xie H., Liu D., Zhao Q., Li Q., Zhang J. (2014). State of the art of single-walled carbon nanotube synthesis on surfaces. Adv. Mater..

[8] Das D., Roy A. (2020). Synthesis of diameter controlled multiwall carbon nanotubes by microwave plasma-CVD on low-temperature and chemically processed Fe nanoparticle catalysts. Appl. Surf. Sci..

[9] Dini Y., Faure-Vincent J., Dijon J. (2019). How to overcome the electrical conductivity limitation of carbon nanotube yarns drawn from carbon nanotube arrays. Carbon.

[10] Kuramochiab E. (2016). Manipulating and trapping light with photonic crystals from fundamental studies to practical applications. J. Mater. Chem. C.

[11] Corletto A., Shapter J.G. (2021). Nanoscale patterning of carbon nanotubes: Techniques, applications, and future. Adv. Sci..

[12] Li J., Papadopoulos C., Xu J.M. (1999). Highly-ordered carbon nanotube arrays for electronics applications. Appl. Phys. Lett..

[13] Su J.S., Lee J.S. (1999). Highly ordered two-dimensional carbon nanotube arrays. Appl. Phys. Lett..

[14] Teo K.B.K., Chhowalla M., Amaratunga G.A.J., Milne W.I., Hasko D.G., Pirio G., Legagneux P., Wyczisk F., Pribat D. (2001). Uniform patterned growth of carbon nanotubes without surface carbon. Appl. Phys. Lett..

[15] Butler T.P., Rashid I., Montelongo Y., Amaratungaa G.A.J., Butt H. (2018). Optical bandgap modelling from the structural arrangement of carbon nanotubes. Nanoscale.

[16] Rajasekharan R., Butt H., Dai Q., Wilkinson T.D., Amaratunga G.A.J. (2012). Can nanotubes make a lens array?. Adv. Mater..

[17] Elmera J.W., Yaglioglua O., Schaefferb R.D., Kardosb G., Derkach O. (2012). Direct patterning of vertically aligned carbon nanotube arrays to 20 μm pitch using focused laser beam micromachining. Carbon.

[18] Labunov V., Prudnikava A., Bushuk S., Filatov S., Shulitski B., Tay B.K., Shaman Y., Basaev A. (2013). Femtosecond laser modification of an array of vertically aligned carbon nanotubes intercalated with Fe phase nanoparticles. Nanoscale Res. Lett..

[19] Wu X., Yin H., Li Q. (2019). Ablation and patterning of carbon nanotube film by femtosecond laser irradiation. Appl. Sci..

[20] Yoon J.-W., So H.-M., Cho S.-H., Chang W.S. (2013). Effect of polarization of ultrafast laser irradiation on carbon nanotube film. Thin Solid Films.

[21] Choi S.B., Byeon C.C., Park D.J., Jeong M.S. (2016). Polarization-selective alignment of a carbon nanotube film by using femtosecond laser ablation. J. Korean Phys. Soc..

[22] Langheinrich D., Dörfler S., Althues H., Kaskel S., Lasagni A. (2012). Rapid and scalable method for direct and indirect microstructuring of vertical aligned carbon nanotubes. Surf. Coat. Technol..

[23] Cheong F.C., Lim K.Y., Sow C.H., Lin J., Ong C.K. (2003). Large area patterned arrays of aligned carbon nanotubes via laser trimming. Nanotechnology.

[24] Gerasimenko A.Y., Kitsyuk E.P., Kuksin A.V., Ryazanov R.M., Savitskiy A.I., Savelyev M.S., Pavlov A.A. (2019). Influence of laser structuring and barium nitrate treatment on morphology and electrophysical characteristics of vertically aligned carbon nanotube arrays. Diamond Relat. Mater..

[25] Lasagni A., Cross R., Graham S., Das S. (2009). The fabrication of high aspect ratio carbon nanotube arrays by direct laser interference patterning. Nanotechnology.

[26] Kichambare P.D., Chen L.C., Wang C.T., Ma K.J., Wu C.T., Chen K.H. (2001). Laser irradiation of carbon nanotubes. Mater. Chem. Phys..

[27] Gbordzoe S., Yarmolenko S., Kanakaraj S., Haase M.R., Alvarez N.T., Borgemenke R., Adusei P.K., Shanov V. (2017). Effects of laser cutting on the structural and mechanical properties of carbon nanotube assemblages. Mater. Sci. Eng. B.

[28] Lim K.Y., Sow C.H., Lin J., Cheong F.C., Shen Z.X., Thong J.T.L., Chin K.C., Wee A.T.S. (2003). Laser pruning of carbon nanotubes as a route to static and movable structures. Adv. Mater..

[29] Zhang L., Li H., Yue K.-T., Zhang S.-L., Wu X., Zi J., Shi Z., Gu Z. (2002). Effects of intense laser irradiation on Raman intensity features of carbon nanotubes. Phys. Rev. B.

[30] Lim Z.H., Lee A., Zhu Y., Lim K.-Y., Sow C.-H. (2009). Sustained laser induced incandescence in carbon nanotubes for rapid localized heating. Appl. Phys. Lett..

[31] Mialichi J.R., Brasil M.J.S.P., Iikawa F., Veríssimo C., Moshkalev S.A. (2013). Laser irradiation of carbon nanotube films: Effects and heat dissipation probed by Raman spectroscopy. J. Appl. Phys..

[32] Kumanek B., Janas D. (2019). Thermal conductivity of carbon nanotube networks: A review. J. Mater. Sci..

[33] Vanab H.H., Badurab K., Zhang M. (2015). Laser-induced transformation of freestanding carbon nanotubes into graphene nanoribbons. Nanoscale.

[34] Momma C., Chichkov B.N., Nolte S., Alvensleben F., Tiinnermann A., Welling H., Wellegehausen B. (1996). Short-pulse laser ablation of solid targets. Opt. Commun..

[35] Okotrub A.V., Bulusheva L.G., Kudashov A.G., Belavin V.V., Komogortsev S.V. (2008). Arrays of carbon nanotubes aligned perpendicular to the substrate surface: Anisotropy of structure and properties. Nanotechnol. Russia.

[36] Gorokhov G.V., Bychanok D.S., Kuzhir P.P., Gorodetskiy D.V., Kurenya A.G., Sedelnikova O.V., Bulusheva L.G., Okotrub A.V. (2020). Creation of metasurface from vertically aligned carbon nanotubes as versatile platform for ultra-light THz components. Nanotechnology.

[37] Gorodetskiy D.V., Kurenya A.G., Gusel’nikov A.V., Baskskova K.I., Smirnov D.A., Arkhipov V.E., Bulusheva L.G., Okotrub A.V. (2020). Laser beam patterning of carbon nanotube arrays for the work of electron field emitters in technical vacuum. Mater. Sci. Eng. B..

[38] Gries W.H. (1996). A Universal Predictive Equation for the Inelastic Mean Free Pathlengths of X-ray Photoelectrons and Auger Electrons. Surf. Interface Anal..

[39] Barinov A., Malcioglu O.B., Fabris S., Sun T., Gregoratti L., Dalmiglio M., Kiskinova M. (2009). Initial stages of oxidation on graphitic surfaces: Photoemission study and density functional theory calculations. J. Phys. Chem. C.

[40] Fedoseeva Y.V., Pozdnyakov G.A., Okotrub A.V., Kanygin M.A., Nastaushev Y.V., Vilkov O.Y., Bulusheva L.G. (2016). Effect of substrate temperature on the structure of amorphous oxygenated hydrocarbon films grown with a pulsed supersonic methane plasma flow. Appl. Surf. Sci..

[41] Zhan D., Ni Z., Chen W., Sun L., Luo Z., Lai L., Yu T., Wee A.T.S., Shen Z. (2011). Electronic structure of graphite oxide and thermally reduced graphite oxide. Carbon.

[42] Laikhtman A., Gouzmna I., Hoffman A., Comtet G., Hellner L., Dujardin G. (1999). Sensitivity of near-edge x-ray absorption fine structure spectroscopy to ion beam damage in diamond films. J. Appl. Phys..

[43] Tang Y.H., Sham T.K., Hu Y.F., Lee C.S., Lee S.T. (2002). Near-edge X-ray absorption fine structure study of helicity and defects in carbon nanotubes. Chem. Phys. Lett..

[44] Wesner D., Krummacher S., Carr R., Sham T.K., Strongin M., Eberhart W., Weng S.L., Willaims G., Howells M., Kampas F. (1983). Synchrotron-radiation studies of the transition of hydrogenated amorphous carbon to graphitic carbon. Phys. Rev. B.

[45] Gorodetskyi D.V., Gusel’nikov A.V., Kerenya A.G., Smirnov D.A., Bulusheva L.G., Okotrub A.V. (2020). Hydrogen Plasma Treatment of Aligned Multi-Walled Carbon Nanotube Arrays for Improvement of Field Emission Properties. Materials.

[46] Sedelnikova O.V., Gurova O.A., Makarova A.A., Fedorenko A.D., Nikolenko A.D., Plyusnin P.E., Arenal R., Bulusheva L.G., Okotrub A.V. (2020). Light-Induced Sulfur Transport inside Single-Walled Carbon Nanotubes. Nanomaterials.

[47] Fedorovskaya E.O., Bulusheva L.G., Kerenya A.G., Asanov I.P., Rudina N.A., Funtov K.O., Lyubutin I.S., Okotrub A.V. (2014). Supercapacitor performance of vertically aligned multiwall carbon nanotubes produced by aerosol-assisted CCVD method. Electrochim. Acta.

[48] di Leo R.A. (2007). Purity assessment of multiwalled carbon nanotubes by Raman spectroscopy. J. Appl. Phys..

[49] Van H.H., Badura K., Zhang M. (2019). Laser-induced transformation of freestanding carbon nanotubes into graphene nanoribbons. J. Mater. Sci..

[50] Shuba M.V., Paddubskaya A.G., Kuzhir P.P., Slepyan G.Y., Maksimenko S.A., Ksenevich V.K., Buka P., Seliuta D., Kasalynas I., Macutkevic J. (2012). Experimental evidence of localized plasmon resonance in composite materials containing single-wall carbon nanotubes. Phys. Rev. B.

[51] Shuba M.V., Yuko D.I., Kuzhir P.P., Maksimenko S.A., Chigir G.G., Pyalitski A.N., Sedelnikova O.V., Okotrub A.V. (2018). Localized plasmon resonance in boron-doped multiwalled carbon nanotubes. Phys. Rev. B.

[52] Okotrub A.V., Kubarev V.V., Kanygin M.A., Sedelnikova O.V., Bulusheva L.G. (2011). Transmission of terahertz radiation by anisotropic MWCNT/polystyrene composite films. Phys. Status Solidi B.

[53] Bychanok D.S., Shuba M.V., Kuzhir P.P., Maksimenko S.A., Kubarev V.V., Kanygin M.A., Sedelnikova O.V., Bulusheva L.G., Okotrub A.V. (2013). Anisotropic electromagnetic properties of polymer composites containingoriented multiwall carbon nanotubes in respect to terahertz polarizer applications. J. Appl. Phys..

[54] Macutkevic J., Seliuta D., Valusis G., Adomavicius R., Krotkus A., Kuzhir P., Paddubskaya A., Maksimenko S., Kuznetsov V., Mazov I. (2012). Multi-walled carbon nanotubes/PMMA composites for THz applications. Diam. Relat. Mater..

[55] Yiang K., Gu M., Guo Y., Pan X., Mu G. (2009). Effects of carbon nanotube functionalization on the mechanical and thermal properties of epoxy composites. Carbon.

[56] Wei Y., Hu X., Sun Z., Wang P., Qiu P., Liu W. (2018). Influence of graphene oxide with different oxidation levels on the properties of epoxy composites. Compos. Sci. Technol..

[57] Matyushkin Y., Danilov S., Moskotin M., Belosevich V., Kaurova N., Rybin M., Obraztsova E.D., Fedorov G., Gorbenko I., Kachorovskii V. (2020). Helicity-Sensitive Plasmonic Terahertz Interferometer. Nano Lett..

