# Supplementary material for: Laser Patterning of Aligned Carbon Nanotubes Arrays: Morphology, Surface Structure, and Interaction with Terahertz Radiation
Source: Materials (Basel). 2021 Jun 14;14(12):3275. doi: 10.3390/ma14123275 (PMC8231912; doi:10.3390/ma14123275)
Supplement: Supplementary file 1 [file materials-14-03275-s001.zip › materials-1225320-supplementary.pdf]

materials

Supplementary Materials

Laser Patterning of Aligned Carbon Nanotubes Arrays: Morphology, Surface Structure, and Interaction with Terahertz Radiation

Olga V. Sedelnikova, Dmitriy V. Gorodetskiy, Alexander G. Kurenya, Kseniya I. Baskakova, Elena V. Shlyakhova, Anna A. Makarova, Gleb V. Gorokhov, Dzmiry S. Bychanok, Polina P. Kuzhir, Sergey A. Maksimenko, Lyubov G. Bulusheva and Alexander V. Okotrub

Correspondence: o.sedelnikova@gmail.com (O.V.S.); spectrum@niic.nsc.ru (A.V.O.)

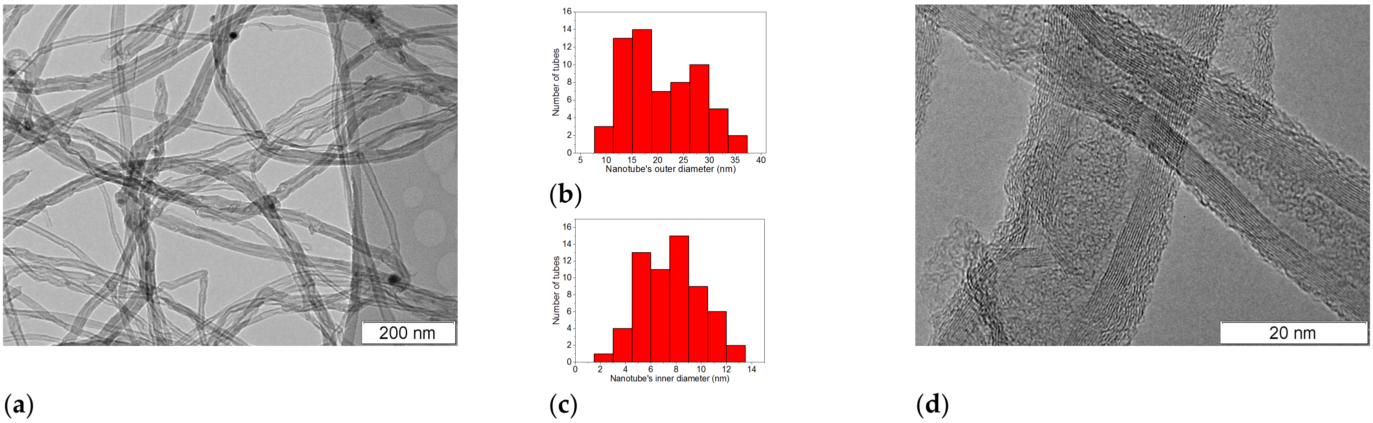

Figure S1. TEM image of MWCNTs from an array (**a**). Outer (**b**) and inner (**c**) diameter distribution of MWCNTs calculated from (**a**). HR TEM image of MWCNTs (**d**).
